# Targeting the Ang2/Tie2 Axis with Tanshinone IIA Elicits Vascular Normalization in Ischemic Injury and Colon Cancer

**DOI:** 10.1155/2021/7037786

**Published:** 2021-11-10

**Authors:** Wei Zou, Cheng Qian, Shan Zhang, Xueting Wan, Zhonghong Wei, Xiaoman Li, Yuanyuan Wu, Wenxing Chen, Aiyun Wang, Yang Zhao, Yin Lu

**Affiliations:** ^1^Jiangsu Key Laboratory for Pharmacology and Safety Evaluation of Chinese Materia Medica, School of Pharmacy, Nanjing University of Chinese Medicine, Nanjing 210023, China; ^2^Jiangsu Collaborative Innovation Center of Traditional Chinese Medicine Prevention and Treatment of Tumor, Nanjing University of Chinese Medicine, Nanjing 210023, China; ^3^Department of Biochemistry and Molecular Biology, School of Medicine & Holistic Integrative Medicine, Nanjing University of Chinese Medicine, Nanjing 210023, China

## Abstract

Pathological angiogenesis, as exhibited by aberrant vascular structure and function, has been well deemed to be a hallmark of cancer and various ischemic diseases. Therefore, strategies to normalize vasculature are of potential therapeutic interest in these diseases. Recently, identifying bioactive compounds from medicinal plant extracts to reverse abnormal vasculature has been gaining increasing attention. Tanshinone IIA (Tan IIA), an active component of *Salvia miltiorrhiza*, has been shown to play significant roles in improving blood circulation and delaying tumor progression. However, the underlying mechanisms responsible for the therapeutic effects of Tan IIA are not fully understood. Herein, we established animal models of HT-29 human colon cancer xenograft and hind limb ischemia to investigate the role of Tan IIA in regulating abnormal vasculature. Interestingly, our results demonstrated that Tan IIA could significantly promote the blood flow, alleviate the hypoxia, improve the muscle quality, and ameliorate the pathological damage after ischemic insult. Meanwhile, we also revealed that Tan IIA promoted the integrity of vascular structure, reduced vascular leakage, and attenuated the hypoxia in HT-29 tumors. Moreover, the circulating angiopoietin 2 (Ang2), which is extremely high in these two pathological states, was substantially depleted in the presence of Tan IIA. Also, the activation of Tie2 was potentiated by Tan IIA, resulting in decreased vascular permeability and elevated vascular integrity. Mechanistically, we uncovered that Tan IIA maintained vascular stability by targeting the Ang2-Tie2-AKT-MLCK cascade. Collectively, our data suggest that Tan IIA normalizes vessels in tumors and ischemic injury via regulating the Ang2/Tie2 signaling pathway.

## 1. Introduction

It has been well recognized that blood vessels mainly including arteries, veins, and capillaries are composed of endothelial cells (ECs), with pericytes and smooth muscle cells to form a complete structure in normal tissues. Nevertheless, tumor blood vessels exhibit atypical morphological characteristics, and the resulting vessels are leaky, immature, thin, and poorly perfused [1, 2]. The highly structurally abnormal and dysfunctional tumor vasculature has been shown to promote tumor progression and induce therapeutic resistance [3]. Vascular abnormalities are not limited to cancer, and they also exist in considerable angiogenic diseases such as ischemic injury. In ischemic diseases, arteriogenesis mediated by blood pressure and shear stress as well as angiogenesis stimulated by hypoxia microenvironment are endogenous [4]. Therefore, vascular morphology is commonly impaired, and these defective vessels cannot participate in the blood circulation system and fail to supply oxygen and nutrients to the ischemic lesions for the recovery [5]. When vascular ECs undergo a tubular shape to form a lumen and then mature into functional vessels, they can provide new oxygenated blood supply for hypoxic tissues [6]. Notably, carbonic anhydrase IX (CA-9), as an intrinsic biomarker of hypoxia, is potently transcriptionally induced and functionally activated under the circumstance of hypoxia [7, 8]. Given the fact that tumors and ischemic diseases share similar pathological features in light of vascular abnormalities, reversing immature and unstable blood vessels back to normal blood vessels appears to be an effective route for the treatments of the diseases.

Vascular normalization refers to partial pruning of immature vessels and remodeling of remaining vessels. It has been well documented that angiopoietin/tyrosine kinase receptor (Ang/Tie) signaling cascade plays a pivotal role in inducing vascular normalization [9]. Ang1 released by pericytes is capable of binding to Tie2 that is predominantly expressed on ECs, contributing to vascular maturation [10]. However, Ang2 is mainly secreted by ECs and acts in an autocrine manner [11]. Although Ang2 is unable to induce Tie2 phosphorylation, it competitively blocks the biological effects of Ang1 that also belongs to Ang family proteins [12]. It was reported that Ang2 inhibition contributed to increased pericyte coverage and basement membrane remodeling, enhanced EC junctions, and uniformed vessel diameter [13]. All of these strengthen the homeostasis of ECs and pericytes as well as their interactions, maintaining the maturation and stabilization of blood vessels.

Tanshinone IIA (Tan IIA), an active component of *Salvia miltiorrhiza*, is known to exert significant effects on improving blood circulation and delaying tumor progression [14, 15]. Although it has been shown that Tan IIA emerges as a promising candidate against ischemic complications and cancer, the underlying mechanisms of its effects still remain elusive, and the function of Tan IIA in these two pathological conditions has not been simultaneously studied in the same animal. In the present study, we demonstrated that Tan IIA triggered vascular normalization in a combined ischemic insult and colon cancer model, which was ascribed to the suppression of Ang2/Tie2 signaling axis. To this end, a better understanding of the multiple functions of Tan IIA may shed new light on the simultaneous clinical management of ischemic injury and colon cancer.

## 2. Materials and Methods

### 2.1. Chemicals and Bioreagents

Tanshinone IIA (purity 98%) was from Jin Yibai Biological Technology Co., Ltd. (Nanjing, China). Roswell Park Memorial Institute (RPMI) 1640 medium, foetal bovine serum (FBS), trypsin-EDTA, and penicillin/streptomycin were obtained from Gibco. The HT-29 cells and human umbilical vein endothelial cells (HUVECs) were purchased from the American Type Culture Collection (ATCC).

### 2.2. Animal Model of HT-29 Xenograft and Hind Limb Ischemia

Male BALB/c nude mice aged 6-8 weeks were purchased from Beijing Vital River Laboratory Animal Technology Co., Ltd. (Beijing, China). Mice were housed under standard laboratory conditions (room temperature: (22 ± 2) °C, humidity (50 ± 5) %, light/dark cycle 12/12 h) and maintained in a specific pathogen-free facility. All experimental protocols were approved by the Animal Care and Use Committee of Nanjing University of Chinese Medicine and conducted in accordance with the Guidelines for the Care and Use of Laboratory Animals (202009A029).

Approximately 2 × 10^6^ HT-29 cells were subcutaneously injected into the right flank region of BALB/c nude mice and allowed to grow for two weeks to develop tumors. When the mean tumor volume reached around 100 mm^3^, the mice were subjected to femoral artery ligation on the left hind limb as previously described [16]. The mice were then randomly divided into four groups (*n* = 8), including model group (daily oral administration of 0.3% CMC-Na at 0.1 ml/10 g of body weight) and three Tan IIA treatment groups (daily oral administration of 10 mg/kg, 30 mg/kg or 90 mg/kg Tan IIA). Tan IIA (Nanjing Jin Yibai Biological Technology Co., Ltd., Nanjing, China) was dissolved in the 0.3% CMC-Na solution. After three weeks of administration, all mice were euthanized by cervical dislocation. The tumors and gastrocnemius muscle tissues were collected and analyzed.

### 2.3. Laser Doppler Perfusion Imaging

The mice were anesthetized with isoflurane, and blood perfusion in both ischemic (left) and nonischemic hind limbs (right) was measured using laser Doppler perfusion imaging (LDPI) (Moor Instruments Ltd, England) before surgery and at different time points (0, 7, 14, and 21 days) following surgery. Blood perfusion ratio was calculated by normalizing the blood perfusion of ischemic hind limbs with that of the contralateral nonischemic hind limbs.

### 2.4. Assessment of Ischemic Damage

The morphologies of ischemic hind limb in mice were assessed at days 7, 14, and 21 and scored as described previously [17]. A similar phenotype of the limb as compared to the nonischemic hind limb was rated as 0. Mild discoloration was scored as 1; moderate discoloration was scored as 2; serve discoloration, subcutaneous tissue loss, or necrosis was scored as 3; and amputation was scored as 4.

### 2.5. Haematoxylin and Eosin (H&E) Staining and Histopathologic Analysis

The gastrocnemius muscle tissues were randomly selected and then fixed with 4% paraformaldehyde (PFA) overnight. The fixed tissues were sequentially washed in 50% methanol for 30 min, 75% methanol for 60 min, 95% methanol for 60 min, 100% methanol for 2 × 60 min, and ethanol/xylene (1 : 1) for 12 h, followed by paraffin embedding. The tissues were then sectioned at 5 *μ*m thickness using a microtome and stained with H&E according to standard protocol. Histological scores were assessed as previously described based on the degree of myofiber atrophy, degenerative necrosis, inflammatory cell infiltration, and calcification [18]. The sum of individual score for the four properties of gastrocnemius muscle ranged from 0 to 12 in each mouse. Pathological examinations and scoring were performed in a double-blinded manner.

### 2.6. Immunofluorescence Staining

The tissues were sectioned at 5 *μ*m thickness and underwent antigen retrieval using citrate buffer solution, or the cells were fixed on a round coverslip and then incubated with indicated primary antibodies overnight at 4°C. The primary antibodies included anti-Dystrophin (12715-1-AP, Proteintech), anti-CD31 (3528, Cell Signaling Technology), anti-Claudin 5 (ab131259, Abcam), anti-PDGFR*β* (3169, Cell Signaling Technology), anti-collagen IV (ab6586, Abcam), anti-ZO-1 (21773-1-AP, Proteintech), and anti-Claudin 1 (13050-1-AP, Proteintech). Alexa Fluor 594 conjugated Goat anti-Rabbit (ab150080, Abcam) and Alexa Fluor 488 conjugated Goat anti-Mouse (ab150113, Abcam) were used as secondary antibodies. Images were acquired with a fluorescence microscope (Zeiss, Oberkochen, Germany).

### 2.7. Immunohistochemistry

The immunohistochemistry assay for hypoxia was performed using CA-9 (11071-1-AP, Proteintech) antibody as previously described. The tissues were harvested and sectioned at 5 *μ*m thickness. After antigen retrieval with citrate buffer (0.01 ml, pH 6.0), the sections were incubated with CA-9 primary antibody at 4°C for 12 h. The sections were washed three times in phosphate-buffered saline (PBS) and then incubated with horseradish peroxidase- (HRP-) labeled anti-rabbit IgG antibody (Bioworld, USA) for 30 min at room temperature, followed by washing with PBS and developing with diaminobenzidine. The images were acquired by Mantra Quantitative Pathology Workstation (PerkinElmer, USA), and the expression levels of proteins were analyzed with Image J software (NIH, Bethesda, USA).

### 2.8. Measurement of Tetramethylrhodamine Isothiocyanate (TRITC)-Dextran Leakage

TRITC-dextran leakage was performed as previously described [19]. Briefly, the mice received an intravenous administration of 100 *μ*l TRITC-dextran (70KDa, Sigma) at a concentration of 25 mg/ml. After 30 min, the tumor tissues were collected, fixed with 4% PFA for 24 h, and sectioned at 5 *μ*m thickness. The tissue sections were then immunostained with the CD31 antibody. The tissue images were acquired with a digital microscope (3D HISTECH Kft, Hungary).

### 2.9. Wound Healing Assay

The wound healing assay was performed as previously described [20]. Briefly, HUVECs (1 × 10^6^ cells/well) were seeded into 6-well plates and grown to 70-80% confluence. After starvation in the serum-free medium for 12 h, PBS was used to wash the HUVECs for 3 times. The confluent monolayer was then wounded by a sterile pipette tip, followed by replacing with the basal medium containing different concentrations of Tan IIA or dimethyl sulfoxide (DMSO). Cells migrating into the wound were photographed under a ZEISS microscope at 0 h and 24 h following scraping at 3-4 different locations. The wound closure was measured by ImageJ 1.5.

### 2.10. Endothelial Monolayer Permeability Assay

1 × 10^6^ HUVECs in 200 *μ*l 1640 media were seeded into the upper chamber of each Transwell (Corning), and 1 ml of 1640 media was added to the bottom chamber. The HUVECs were incubated at 37°C overnight to allow the formation of a uniform dense monolayer, after which the medium in the upper chamber was aspirated carefully and replaced with the medium containing 200 ng/ml Ang2 or Ang2 (a final concentration of 200 ng/ml) plus different concentrations of Tan IIA. After incubation for 24 h, 0.1 mg/ml fluorescein isothiocyanate- (FITC-) dextran (40 kDa, Sigma) was added into the upper chamber, and the HUVECs were incubated at 37°C for 90 min. Subsequently, 100 *μ*l medium was collected from the bottom chamber of each well and then added into a 96-well black plate. Fluorescence values for all samples were measured using a PerkinElmer Victor V fluorescence plate reader (exiation: 480 nm, emission: 520 nm).

### 2.11. Enzyme-Linked Immunosorbent Assay (ELISA)

The Ang2 ELISA Kit (Nanjing Jin Yibai Biological Technology Co., Ltd., Nanjing, China) was used to detect the levels of Ang2 in either mouse serum samples or cell culture supernatants. The ELISA assay was performed according to the manufacturer's instructions.

### 2.12. RNA Isolation and Real-Time PCR

The HT-29 tumors and gastrocnemius muscle tissues were snap-frozen with liquid nitrogen after resection. Next, the total RNA was extracted using RNAiso Plus reagent (Takara, Japan) according to the manufacturer's instructions. cDNA was synthesized from 500 ng of total RNA using Hiscript®II QRT SuperMix (Vazyme, Shanghai, China). Real-time PCR was performed using ChamQ SYBR qPCR Master Mix (Low ROX Premixed) (Vazyme, Shanghai, China) and detected by ABI 7500 system (Applied Biosystems, CA, USA). The sequence of primers used in this study can be found in Table S1.

### 2.13. Western Blotting

Cellular or tumor tissue homogenates were lysed in the radioimmunoprecipitation assay (RIPA) buffer (Thermo Fisher Scientific, Waltham, MA, USA) containing protease and phosphatase inhibitors (Roche, Cat. No.: 04693116001, 04906837001) on ice for 30 min. After centrifugation at 14000 rpm for 5 min at 4°C, the supernatant was collected, and the protein concentration was determined using the BCA protein assay kit. Western blotting was performed as previously described [21]. Briefly, the total proteins were separated by sodium dodecyl sulphate-polyacrylamide gel electrophoresis and immunoblotted with indicated antibodies against Phospho-Tie2 (Tyr992) (4221, Cell Signaling Technology), Phospho-AKT (serS473) (4060, Cell Signaling Technology), Phospho-Myosin Light Chain 2 (Ser19) (3671, Cell Signaling Technology), ZO-1 (21773-1-AP, Proteintech), Claudin 1 (13050-1-AP, Proteintech), VE-cadherin (Sc-9989, Santa Cruz), and GAPDH (AP0063, Bioworld). The protein bands were visualized with enhanced chemiluminescence (ECL) reagent (Biosharp, Wuhan, China), and the expression of proteins was quantified based on scanning densitometry (Gel Doc-2000, Bio-Rad).

### 2.14. Statistical Analysis

All data were presented as mean ± standard deviation (SD). The data were analyzed by one-way analysis of variance (ANOVA) for multiple comparisons. *p* < 0.05 was considered statistically significant.

## 3. Results

### 3.1. Tan IIA Improved Blood Perfusion Recovery in the Ischemic Hind Limbs

**Figure 1:**
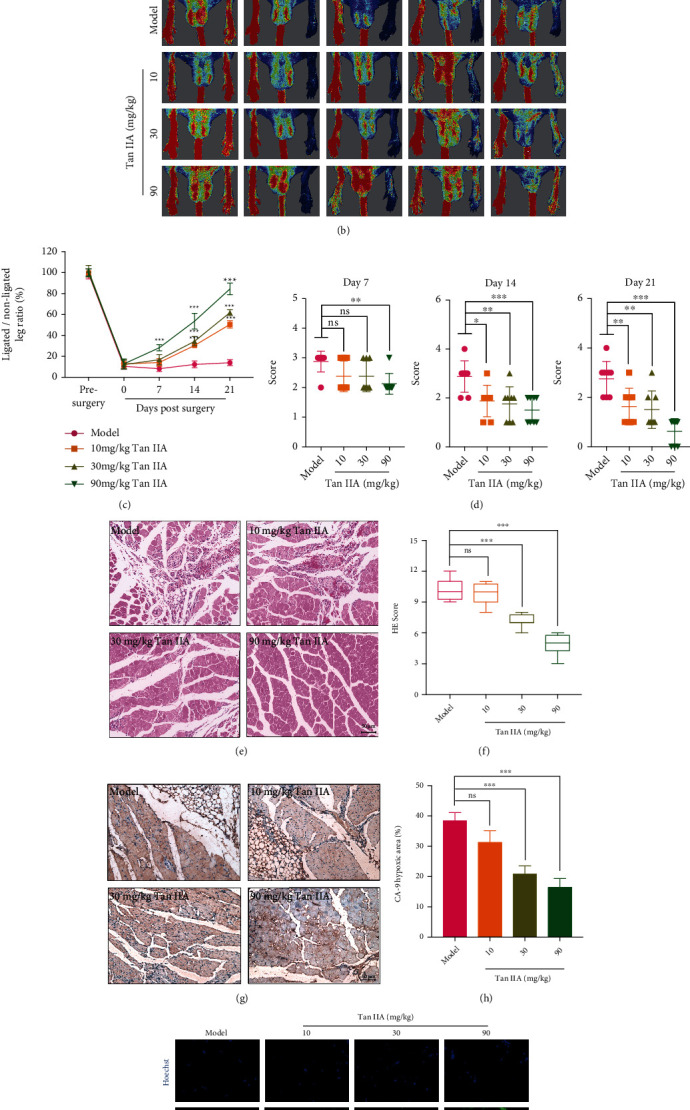
Tan IIA improved blood perfusion recovery in the ischemic hind limbs. (a) Schematic diagram for depicting the establishment of the combined mouse model of HT-29 xenograft and hind limb ischemia, including the schedule of Tan IIA treatments. (b) Representative images of LDPI of the ischemic hind limbs in mice treated with 0.3% CMC-Na or Tan IIA at the indicated time points. (c) Hindlimb blood flow expressed as a percentage of ischemic limb blood flow over nonischemic hindlimb blood flow measured at the indicated time points (*n* = 6). (d) Morphology assessment of ischemic hind limbs in mice treated with 0.3% CMC-Na or Tan IIA at the indicated time points (*n* = 8). (e) Representative images of H&E staining for the gastrocnemius muscle at 21 days postsurgery. Scale bar, 50 *μ*m. (f) Histological scoring of H&E staining for the mice treated with 0.3% CMC-Na or Tan IIA (*n* = 8). (g) Hypoxia in the gastrocnemius muscle tissues at day 21 postsurgery was measured by CA-9 staining (brown). Representative images are shown. Scale bar, 50 *μ*m. (h) Statistical analysis of CA-9 expression in the gastrocnemius muscle tissues (*n* = 3). (i) Representative immunofluorescence images of Dystrophin (green) to reflect functional muscle fibers in the gastrocnemius muscle tissues are shown. Scale bar, 25 *μ*m. The data were presented as mean ± SD. ^∗^*p* < 0.05, ^∗∗^*p* < 0.01, ^∗∗∗^*p* < 0.001 (versus model group).

To evaluate the effects of Tan IIA on influencing the alterations in blood vessels of different pathological tissues, BALB/c nude mice were utilized to establish a combined mouse model of hind limb ischemia and HT-29 xenograft (Figure 1(a)). We first examined the role of Tan IIA in blood perfusion recovery in the ischemic hind limbs by a LDPI system. It was shown that intragastric administration of Tan IIA resulted in blood perfusion recovery starting from day 7 postsurgery. Indeed, Tan IIA-treated mice displayed a significantly higher blood perfusion recovery at day 21 postsurgery (10 mg/kg: 50.55%; 30 mg/kg: 61.88%; 90 mg/kg: 84.47%) compared to the mice received 0.3% CMC-Na (model group) (Figures 1(b) and 1(c)). In the assessment of ischemic damage, our results showed that the mice treated with Tan IIA did not exhibit obvious necrosis, and the score was between 0 and 2 while the mice treated with 0.3% CMC-Na were scored 2–4 at day 21 postsurgery (Figure 1(d)). Of note, the endogenous neovascularization mediated by high blood pressure and shear stress was frequently lack of lumen, and it thus might not be effectively involved in the blood circulation in the ischemic tissues [22]. The sustained hypoxia was responsible for failure of muscle recovery, extensive tissue necrosis, and severe muscle mass loss [23]. As shown in Figures 1(e) and 1(f), the ischemic hind limbs of the mice in the model group presented the phenotypes of muscle fiber degeneration, necrosis, inflammation, and calcification, which could be rescued by 30 and 90 mg/kg of Tan IIA. Immunohistochemistry results revealed that the gastrocnemius from Tan IIA-treated (30 and 90 mg/kg) mice displayed decreased CA-9 expression compared to those of mice in the model group (Figures 1(g) and 1(h)). Furthermore, the expression of dystrophin was substantially boosted in the ischemic hind limbs of Tan IIA-treated mice than those of the model mice (Figure 1(i)). In order to determine whether the two models on the same mice affected each other, we thus established the hind limb ischemia mice model and HT-29 human colon cancer xenograft mouse model, respectively. This enabled us to study the effects of Tan IIA on separate models. In the ischemic hind limbs model, it was shown that Tan IIA could significantly promote the blood perfusion (Supplementary Figure 1A and 1B), improve the muscle quality (Supplementary Figure 1C and 1D), alleviate the hypoxia (Supplementary Figure 1E and 1F), and ameliorate the pathological damage (Supplementary Figure 1G and 1H). Together, our results deciphered that intragastric administration of Tan IIA could strikingly reinforce the blood perfusion recovery, which was potentially owing to the improved formation of functional blood vessels in the ischemic hind limbs.

### 3.2. Tan IIA Induced Normalization of Tumor Blood Vessels

**Figure 2:**
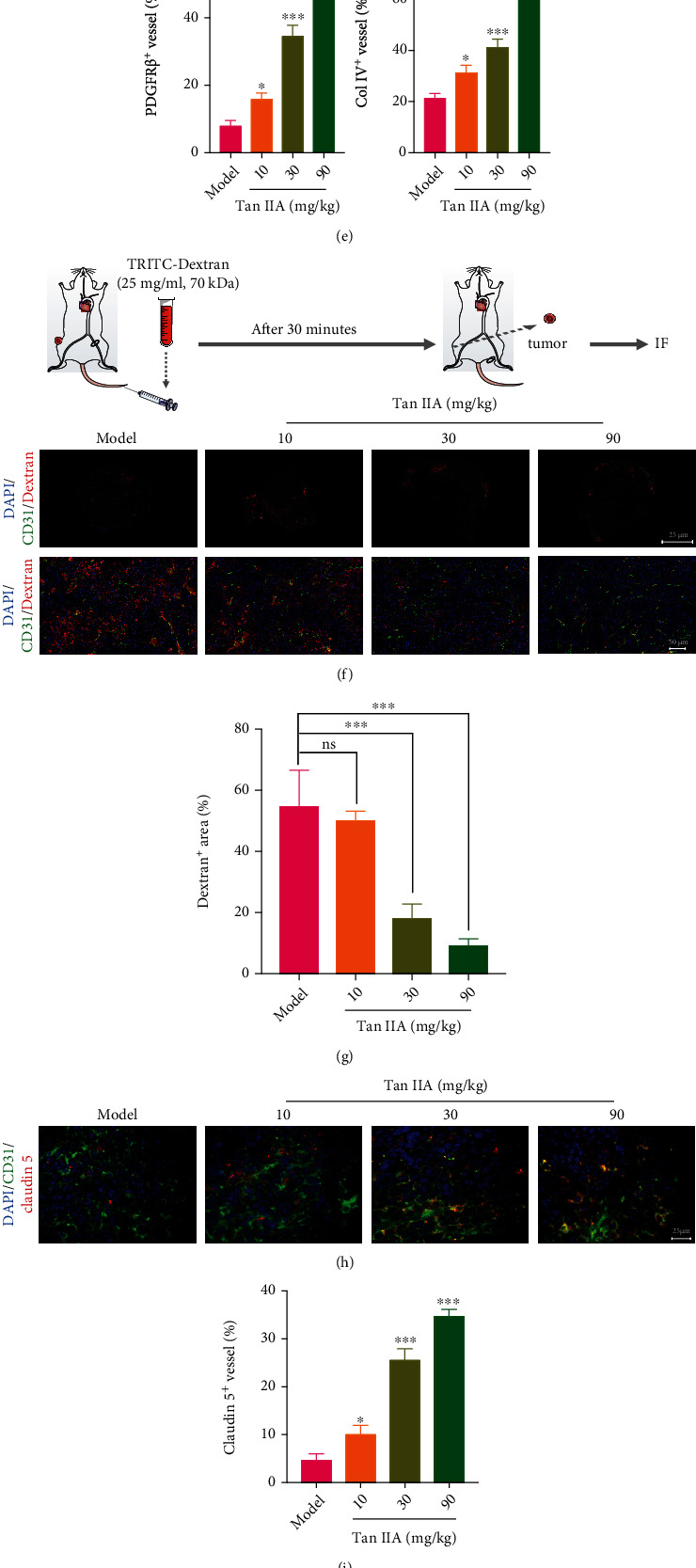
Tan IIA induced normalization of tumor blood vessels. (a) Growth curve of HT-29 tumors in the mice treated with 0.3% CMC-Na and Tan IIA (10 mg/kg, 30 mg/kg, and 90 mg/kg) (*n* = 8). (b) Representative picture of HT-29 tumors harvested from mice treated with 0.3% CMC-Na and Tan IIA at day 21 postsurgery (*n* = 8). (c) Tumor weights from different groups of mice were measured on day 21 postsurgery (*n* = 8). (d) Representative immunofluorescence images of PDGFR*β* and collagen IV in the tumor blood vessels are shown. Scale bars, 25 *μ*m. (e) Quantification of PDGFR*β* and collagen IV expression in the tumor blood vessels (*n* = 3). (f) Representative images of tumor vascular leakiness in the mice treated with 0.3% CMC-Na and Tan IIA. TRITC-dextran was intravenously injected into BALB/c nude mice bearing HT-29 tumors. The extravasated TRITC-dextran from tumor blood vessels stained for CD31 is shown. (g) The TRITC-dextran leakage was quantified by the ratios of dextran^+^ area to CD31^+^ area (*n* = 3). Scale bars, 50 *μ*m. (h) Representative immunofluorescence images of Claudin 5 in the tumor blood vessels are shown. Scale bars, 25 *μ*m. (i) Quantification of Claudin 5 expression in the tumor blood vessels (*n* = 3). (j) Hypoxia in the tumor parenchyma was determined by CA-9 staining (brown) at day 21 postsurgery. Representative immunohistochemical staining images are shown. Scale bar, 25 *μ*m. (k) Quantification of CA-9 expression in the HT-29 tumors harvested from the mice treated with 0.3% CMC-Na and Tan IIA (*n* = 3). The data were presented as mean ± SD. ^∗^*p* < 0.05, ^∗∗^*p* < 0.01, ^∗∗∗^*p* < 0.001 (versus model group).

To further explore the function of Tan IIA in regulating tumor blood vessels, HT-29 colorectal cancer cells were also subcutaneously injected into the BALB/c nude mice and allowed to develop tumors. Surprisingly, our results unveiled that Tan IIA significantly impaired tumor growth, as evidenced by the fact that the relative tumor volumes in the mice treated with Tan IIA (30 mg/kg and 90 mg/kg) were significantly lower than those of mice treated with 0.3% CMC-Na on day 21 postsurgery (Figures 2(a) and 2(b)). Consistently, 10 mg/kg, 30 mg/kg, and 90 mg/kg of Tan IIA treatment led to a significant decrease in the average tumor weight compared with 0.3% CMC-Na treatment on day 21 postsurgery (Figure 2(c)). In addition, Tan IIA did not cause obvious side effects, as visualized by the normal weight and visceral index of mice (Supplementary Figure 2A and 2B). Given that abnormal angiogenesis plays a vital role in accelerating tumor progression, we further analyzed the tumor vascular structure and function following the treatments of Tan IIA. It was shown that the pericyte coverage and basement membrane support were both remarkably elevated [24] upon Tan IIA treatments, as validated by the increased expression of platelet-derived growth factor receptor *β* (PDGFR*β*) and collagen type IV (Col IV) in the tumor blood vessels, respectively (Figures 2(d) and 2(e)). These data indicated that Tan IIA was able to fortify tumor vascular structure. In light of tumor vascular structure, we demonstrated that Tan IIA contributed to reduced vascular permeability and enhanced vascular integrity, as reflected by the diminished leakage of TRITC-dextran dye (Figures 2(f) and 2(g)). This was substantiated by the fact that the expression levels of claudin 5 as the critical hub of tight junctions were dramatically enhanced in the presence of Tan IIA (10, 30 and 90 mg/kg) (Figures 2(h) and 2(i)). Further, it was observed that Tan IIA markedly reduced the protein expression of CA-9 in the tumor parenchyma, implying that the hypoxia microenvironment was ameliorated with the achievement of vascular normalization (Figures 2(j) and 2(k)). Likewise, in the HT-29 tumor model alone, Tan IIA treatment contributed to impaired tumor development (Supplementary Figure 3A). The tumor vascular integrity was significantly boosted in response to Tan IIA, as shown by the increased pericyte coverage (Supplementary Figure 3B and 3C), basement membrane support (Supplementary Figure 3B and 3D), and tight junctions (Supplementary Figure 3B and 3E). Also, tumor hypoxia was potently reduced following the treatment of Tan IIA (Supplementary Figure 3F and 3G). Collectively, tumor vascular normalization was achieved upon Tan IIA treatment.

### 3.3. Tan IIA Resulted in the Repression of Circulating Ang2 Levels

**Figure 3:**
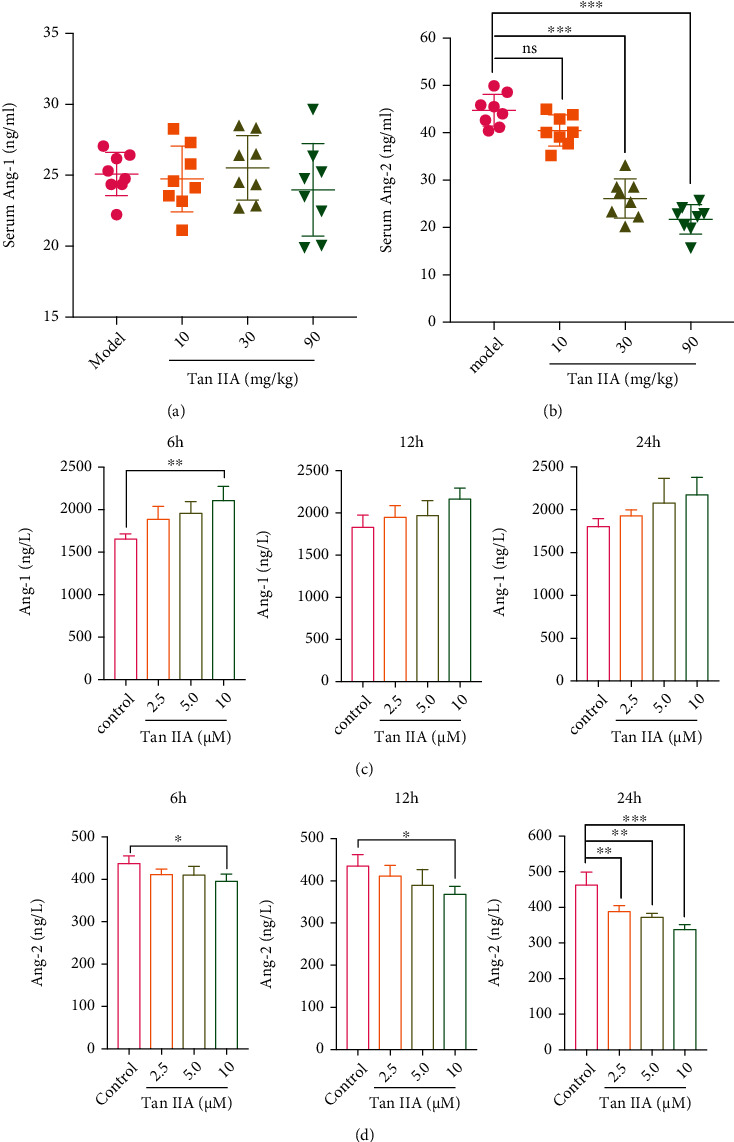
Tan IIA resulted in the repression of circulating Ang2 levels. (a) The Ang1 levels in the serum of mice treated with 0.3% CMC-Na and Tan IIA at day 21 postsurgery (*n* = 8). (b) The Ang2 levels in the serum of mice treated with 0.3% CMC-Na and Tan IIA at day 21 postsurgery (*n* = 8). (c) The Ang1 levels in the supernatant of HUVECs at 6 h, 12 h, and 24 h after DMSO or Tan IIA treatments (*n* = 3). (d) The Ang2 levels in the supernatant of HUVECs at 6 h, 12 h, and 24 h after DMSO or Tan IIA treatments (*n* = 3). The data were presented as mean ± SD. ^∗^*p* < 0.05, ^∗∗^*p* < 0.01, ^∗∗∗^*p* < 0.001 (versus model group).

The Ang/Tie signaling cascade has emerged as a major regulator of vascular homeostasis and a promising therapeutic target [25, 26]. In numerous vascular leak-associated diseases, the concentrations of angiopoietins are rigorously skewed in favor of Ang2, which aggravates the disease progression through triggering vessel permeability and instability [27]. Since Tan IIA was able to restore blood perfusion in the ischemic hind limbs and repair the abnormal tumor vasculature, and we tended to explore if Ang2 inhibition was involved in the pharmacological effects of Tan IIA. To this end, the levels of circulating Ang1 and Ang2 in mice bearing hind limb ischemia and HT-29 tumors were monitored by ELISA assay. To our surprise, there were no significant differences in the levels of circulating Ang1 between model and Tan IIA treated groups (Figure 3(a)). Nevertheless, Tan IIA mitigated the levels of Ang2 in a dose-dependent manner, with 30 mg/kg and 90 mg/kg of Tan IIA treated groups presenting significant difference compared to the model group (Figure 3(b)). Owing to the fact that Ang2 is mainly secreted by ECs, the supernatant of HUVECs was collected at 6 h, 12 h, and 24 h following Tan IIA treatments to examine the levels of Ang1 and Ang2. It was illustrated that 10 *μ*M of Tan IIA promoted the secretion of Ang1 from HUVECs at 6 h, but not 12 h and 24 h (Figure 3(c)). Inversely, the secretion levels of Ang2 from HUVECs were significantly decreased in response to Tan IIA (2.5 *μ*M, 5 *μ*M, and 10 *μ*M) treatments at 24 h (Figure 3(d)). Thus, these results suggested that Tan IIA could significantly inhibit the levels of circulating Ang2 both in vitro and in vivo.

### 3.4. Tan IIA Decreased EC Permeability via Inhibiting Ang2-Mediated Signaling Cascade

**Figure 4:**
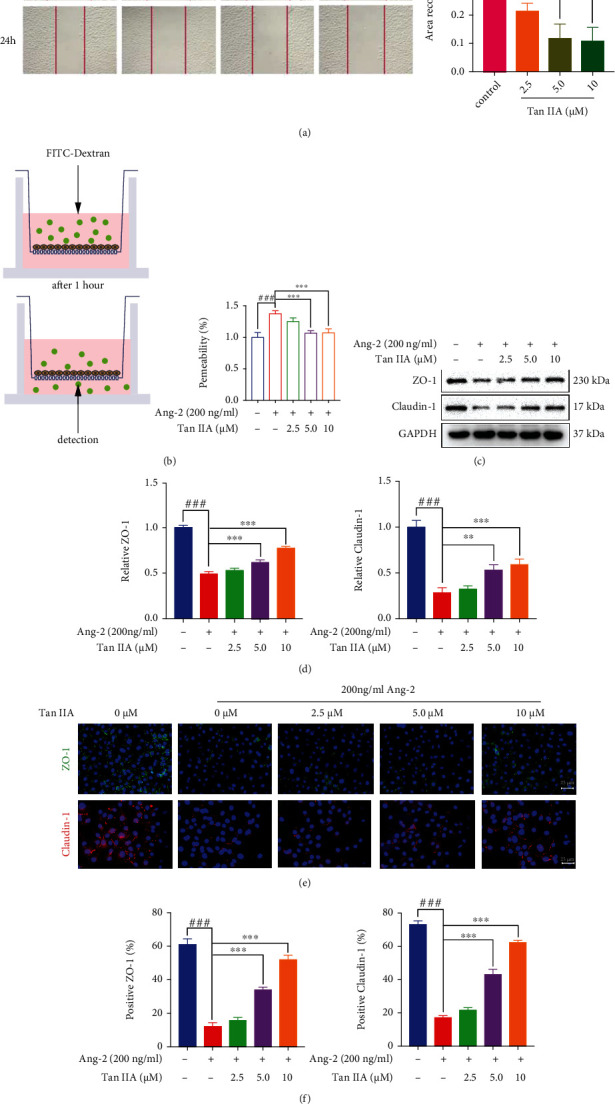
Tan IIA decreased EC permeability via inhibiting Ang2-mediated signaling cascade. (a) The HUVECs were treated with various concentrations of Tan IIA (0, 2.5, 5, and 10 *μ*M) for 24 h. The area covered by migrating HUVECs was photographed by a phase-contrast microscopy. The migration of HUVECs was assessed on the basis of the wound closure area (*n* = 3). (b) Permeability measured in DMSO or Tan IIA treated HUVECs in the absence or presence of Ang2. The HUVEC permeability was quantified by the fluorescence of FITC-dextran (40 kD) collected in the bottom chamber (*n* = 3). (c) The expression of ZO-1 and Claudin-1 in the lysates of HUVEC treated with various concentrations of Tan IIA with or without the stimulation of 200 ng/ml of Ang2. GAPDH was used as a loading control. (d) Changes in the levels of ZO-1 and Claudin-1 were measured as pixel density and normalized to GAPDH (*n* = 3). (e) Representative immunofluorescence images of ZO-1 (green) and Claudin-1 (red) in HUVECs treated various concentrations of Tan IIA in the absence or presence of 200 ng/ml of Ang2. (f) Quantification of ZO-1 and Claudin-1 expression (*n* = 3). The data were presented as mean ± SD. ^∗^*p* < 0.05, ^∗∗^*p* < 0.01, ^∗∗∗^*p* < 0.001 (versus control).

Ang2 levels are elevated in ECs during pathological angiogenesis, leading to the impairment of EC stability and integrity in an autocrine manner [28]. Accordingly, Ang2-mediated downstream signaling acts as a fundamental modulator to give rise to EC dysfunction. Hence, we next investigated the impacts of Tan IIA on regulating Ang2-EC biological behaviors and the expression of EC junctions-associated proteins. In the wound healing assay, it was observed that the migration of HUVECs was significantly prohibited following 24 h treatments of various concentrations of Tan IIA (2.5 *μ*M, 5 *μ*M, and 10 *μ*M) (Figure 4(a)). Further, the role of Tan IIA in governing endothelial integrity was assessed by the transendothelial diffusion of FITC-dextran (40 kD) across the HUVEC monolayers that seeded into Transwell supports. Interestingly, 200 ng/ml of Ang2 alone resulted in a significant increase in the leakage of FITC-dextran into the lower chamber. However, 5 *μ*M and 10 *μ*M of Tan IIA appeared to reverse Ang2-induced EC permeability, as evidenced by decreased FITC-dextran leakage following the treatments of Tan IIA in the presence of Ang2 (Figure 4(b)). To further elucidate the routes by which Tan IIA rescued Ang2-induced EC permeability, the expressions of a series of tight junctions-associated proteins were thus examined by western blot. Notably, the protein expressions of ZO-1 and Claudin-1, the well-known proteins associated with tightening intercellular junctions, were strikingly downregulated after the stimulation of Ang2, which could be rescued in the presence of 5 *μ*M and 10 *μ*M of Tan IIA (Figures 4(c) and 4(d)). In agreement of western blot results, immunofluorescence staining data revealed that the impaired levels of ZO-1 and Claudin-1 after the stimulation of Ang2 could be boosted in response to Tan IIA (Figures 4(e) and 4(f)).

### 3.5. Tan IIA Rescued the Disrupted Tie2 Activation Mediated by Ang2

**Figure 5:**
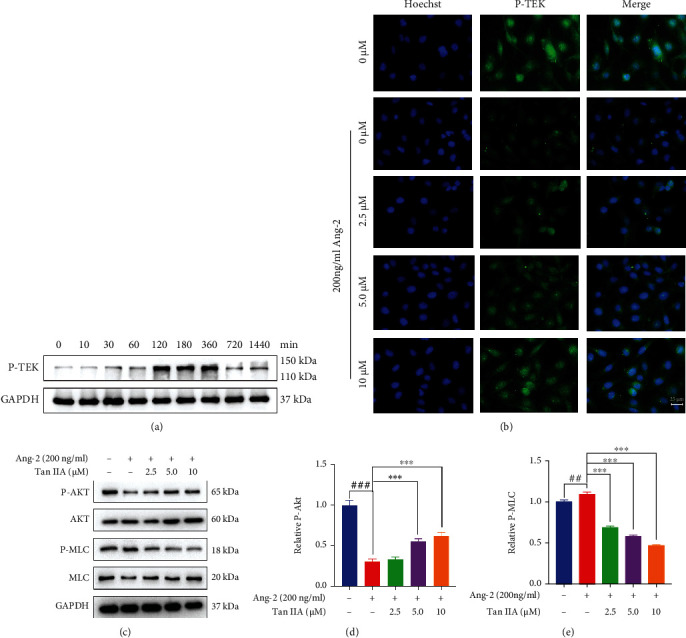
Tan IIA rescued the disrupted Tie2 activation mediated by Ang2. (a) Phosphorylation of Tie2 in HUVEC lysates 24 h after the treatment of 10 *μ*M Tan IIA. GAPDH was used as a loading control (*n* = 3). (b) Representative immunofluorescence images of phospho-Tie2 (green) in HUVECs treated with various concentrations of Tan IIA in the absence or presence of 200 ng/ml of Ang2 (*n* = 3). (c) Phosphorylation of AKT, MLC, and total AKT, MLC in HUVEC lysates 24 h after the treatment of Tan IIA in the absence or presence of Ang2. GAPDH was used as a loading control. (d) Densitometric ratio for AKT activity was quantified. (e) Densitometric ratio for MLC activity was quantified (*n* = 3). The data were presented as mean ± SD. ^∗^*p* < 0.05, ^∗∗^*p* < 0.01, ^∗∗∗^*p* < 0.001 (versus control).

Given that Ang2 competitively inhibits the action of Ang1 on Tie2 and promotes vascular remodeling by suppression of Tie2 signaling [29], we were inclined to investigate the effects of Tan IIA on the disrupted Tie2 signaling by Ang2. In this regard, the HUVECs were proincubated with 10 *μ*M Tan IIA for different time periods followed by exposure to 200 ng/ml of Ang2, and we found that the phosphorylation level of Tie2 culminated at 2 h following Tan IIA treatment (Figure 5(a)). The results were also validated by immunofluorescence staining images, which exhibited that the phosphorylation of Tie2 was dramatically attenuated in the HUVECs stimulated by Ang2 compared to that in nontreated HUVECs, whereas Tan IIA was capable of strengthening the overall fluorescence intensity of p-Tie2 in a dose-dependent manner after exposure to Ang2 (Figure 5(b)). It has been well known that phosphorylation of Tie2 can activate AKT and Rap1 GTPase signaling pathway, leading to a reduction in the phosphorylation of the downstream motor protein myosin light chain (MLC) [30]. As shown in Figures 5(c) and 5(d), Ang2 alone abolished the phosphorylation of AKT in HUVECs. However, Tan IIA dose-dependently amplified AKT phosphorylation even in the presence of Ang2. In line with this result, the elevated phosphorylation of MLC by Ang2 was diminished by Tan IIA in a dose-dependent manner, though no obvious changes were observed in the expression of total MLC (Figures 5(c) and 5(e)).

### 3.6. Tan IIA Potentiated the Activation of Tie2 Signaling Pathway In Vivo

**Figure 6:**
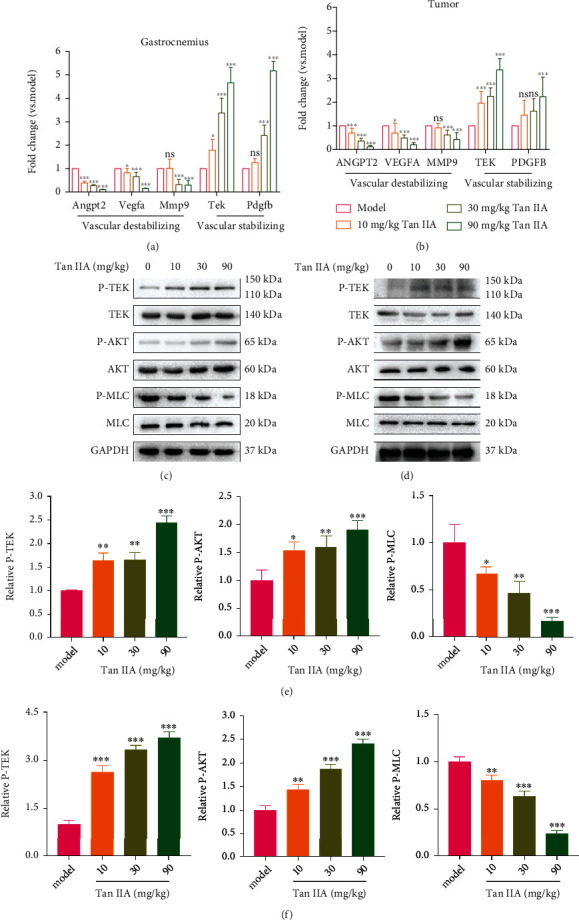
Tan IIA potentiated the activation of Tie2 signaling pathway *in vivo*. (a) The mRNA expressions of indicated genes in the gastrocnemius muscle tissues were measured by real-time PCR (*n* = 8). (b) The mRNA expression of indicated genes in the transplanted HT-29 tumors was measured by real-time PCR (*n* = 8). (c) Phosphorylation of Tie2, AKT, MLC, and total Tie2, AKT, and MLC in the lysates from the gastrocnemius muscle tissues treated with various concentrations of Tan IIA. GAPDH was used as a loading control. (d) Phosphorylation of Tie2, AKT, and MLC and total Tie2, AKT, and MLC in the lysates from the harvested HT-29 tumors treated with various concentrations of Tan IIA. GAPDH was used as a loading control. (e) Densitometric ratios for the activities of Tie2, AKT, and MLC in the lysates from the gastrocnemius muscle tissues were quantified (*n* = 3). (f) Densitometric ratios for the activities of Tie2, AKT, and MLC in the lysates from the harvested HT-29 tumors were quantified (*n* = 3). The data were presented as mean ± SD. ^∗^*p* < 0.05, ^∗∗^*p* < 0.01, ^∗∗∗^*p* < 0.001 (versus model group).

To further investigate whether Tan IIA was able to activate Tie2 signaling pathway *in vivo*, total RNA and proteins were extracted from gastrocnemius and tumor tissues. In gastrocnemius tissues, the mRNA expressions of an array of proangiogenic genes (Angpt2, Vegfa, and Mmp9) were significantly hampered following the treatments of Tan IIA. On the contrary, the mRNA levels of multiple vascular stabilization-related proteins including Tie2 and Pdgfb were strikingly enhanced upon Tan IIA treatments (Figure 6(a)). The similar results in light of mRNA expression changes by Tan IIA were also seen in the transplanted HT-29 tumors (Figure 6(b)). More importantly, in consistent with the *in vitro* data, Tan IIA treatment contributed to dose-dependent increase in the Tie2 and AKT activities, but a decrease in the MLC activity both in the ischemic gastrocnemius (Figures 6(c) and 6(e)) and tumor tissues (Figures 6(d) and 6(f)). These results suggested that Tan IIA might “reprogram” the gene expression pattern, shifting from a proangiogenic profile to a vascular stabilization signature through regulating the Ang2/Tie2 axis.

## 4. Discussion

A growing body of evidence has shown that blood vessels in ischemic injury and tumors are predominantly morphologically immature and functionally unstable, promoting the development and progression of the two diseases [1, 2, 4–6]. To this end, vascular normalization has been proved to act as an effective therapeutic strategy through restoring the organized and functional vascular network [31]. It has been widely held that Ang2 as an antagonistic ligand of the endothelial-specific Tie2 receptor destabilizes blood vessels via competitively antagonizing Ang1 [12]. In this study, we demonstrated that tanshinone IIA, an active component of *Salvia miltiorrhiza*, fortified the vascular structure and enhanced the vascular function through repressing the Ang2/Tie2 signaling axis, resulting in the vascular normalization of tumor and ischemic tissues in a combined mouse model.

In fact, normal blood vessels are in the state of stabilization, with ECs proliferating at a reasonable speed [32]. The connection between ECs is tight, and they collaborate with smooth muscle cells and pericytes to maintain endothelial barrier. However, in ischemic diseases, arteriogenesis mediated by blood pressure and shear stress as well as angiogenesis stimulated by hypoxia microenvironment are endogenous [4, 33], and the organism attempts to use these endogenously formed blood vessels to increase blood supply. Nonetheless, the morphology of blood vessels is usually damaged, and they fail to function like normal blood vessels. These defective blood vessels neither effectively participate in the blood circulatory system nor provide oxygen and nutrients for the recovery of ischemic tissues, resulting in tissue necrosis and loss of muscle mass [6, 34]. Our study found that Tan IIA could significantly promote the recovery of blood flow in the ischemic lower extremity, improve the tissue necrosis, reduce the hypoxia of the gastrocnemius muscle, and enhance muscle mass, indicating that Tan IIA might have significant impacts on the blood vessels in ischemic injury. Since the recovery of blood flow in the ischemic hind limbs is mainly governed by the orderly formed blood vessels and alleviated hypoxic microenvironment, it will be of great interest to explore whether Tan IIA repairs vascular structure and function in the ischemic hind limbs via strengthening the endothelial integrity and reprogram endothelium-associated microenvironment.

In coincident with blood vessel in ischemic hind limbs, tumor blood vessels are characterized by aberrant vascular morphology, with highly proliferative ECs and loosely connected endothelial junctions, giving rise to a striking phenotype of vascular leakage [35]. Tumor blood vessels are not capable of fulfilling the commitment to transport the oxygen, immune cells, and therapeutic agents owing to the permeable profile, which leads to an acidic and hypoxic microenvironment as well as impaired drug delivery and immune cell infiltration [36]. Our data demonstrated that Tan IIA could promote the integrity of tumor vascular structure, as reflected by the boosted pericyte coverage and basement membrane support. However, the increased pericyte coverage is due to improved recruitment of pericytes, or enhanced EC-pericyte interaction is worthy of further investigation. Moreover, we revealed that Tan IIA resulted in reduced tumor vascular leakage and alleviated hypoxia, restoring the tumor vascular function. As a consequence, the normalization of tumor blood vessels triggered by Tan IIA delayed the progression of tumors. Since tumor vascular normalization and immune reprogramming can form a positive feedback loop [37], determining if the penetration of different immune cell subsets can be changed by Tan IIA and can be on the next-to-do list.

Multiple lines of evidence have uncovered that there is a high incidence that tumor and ischemic disease cooccur in the same patient [38, 39]. A range of genes including VEGF, HIF1*α*, ANG, TNF, and mTOR have been reported to participate in the modulation of the formation of blood vessels [40–43]. Ang/Tie signaling axis emerges as the key gatekeeper to influence the process of vascular maturation and remodeling. Ang family is mainly composed of four components including Ang1, Ang2, Ang3, and Ang4, among which Ang1 and Ang2 preferentially bind to Tie2 that is expressed on ECs with similar affinity, but they have opposite regulatory effects on ECs [12]. There have not been many studies on Ang3 and Ang4, and their characteristics and functions are still undefined. Ang1 is predominantly expressed in pericytes and smooth muscle cells; it binds to Tie2 in a paracrine manner to maintain vascular stability and EC survival [10, 44]. However, Ang2 is only expressed in ECs and acts on neighboring ECs in the form of autocrine regulation [11]. It is a competitive antagonist of Ang1 to interact with Tie2 and makes the blood vessels unstable.

The signal transduction of Ang/Tie axis is closely related to cell microenvironment. In inflammatory microenvironment, inflammatory factors can increase the activity of FoxO1, enhance the level of autocrine Ang2, and weaken the signal transduction of Ang1/Tie2 [45]. Excessive release of Ang2 not only inhibited the phosphorylation level of Tie2 and restricted the PI3K signaling pathway [46] but also limited the proliferation and migration of ECs through deactivating the GRB2 signaling pathway [47]. In the context of hypoxia, Cx43 as a Cx isoform was shown to be involved in MEGJ-mediated Ang2 expression in a VEC-dependent manner, which induced iNOS protein levels and vascular hyporeactivity [48], thereby playing a role in vascular regulation. In resting state, Ang1 can bind to Tie2 to activate downstream RAS signaling, reduce cytoskeleton rearrangement, and maintain vascular stability [49]. When stimulated by thrombin, the vascular endothelial barrier will be destroyed [50]. Thus, inflammatory factors, hypoxia, thrombin, and other driving forces can have impacts on the Ang/Tie signaling axis, affecting the stability of blood vessels. In tumor and ischemic tissues, ECs tend to secrete excessive Ang2, resulting in the generation of abnormal blood vessels. In this regard, pursing whether Tan IIA exerts potential effects on the upstream mediators of Ang/Tie signaling may offer additional implications for demonstrating the role of Tan IIA in regulating vascular network.

An increasing number of studies have illustrated that Ang2 is a biomarker for poor prognosis of ischemic diseases [51, 52]. Ang2 is highly expressed in remodeling blood vessels and accelerating the formation of immature blood vessels. Inhibiting Ang2 is of great benefit to the recovery of blood flow [53]. In addition, it was reported that inhibition of Ang2 could significantly reduce tumor growth and vascular density in the Colo205 tumor model that is sensitive to Ang2 inhibition. More importantly, the suppression of Ang2 reversed the tumor blood vessels back to normal blood vessels, as evidenced by the accumulation of critical junctions molecules, increased pericyte coverage, and decreased ECs budding and more uniform vascular distribution [54]. However, manipulation of Ang1 to enhance its expression has no such significant effects on the abnormal vascular system, indicating that inhibition of Ang2 possesses more therapeutic potential than targeting Ang1. Based on the fact that Ang1 and Ang2 can mediate the balance between vascular stabilization and destabilization, more efforts have been devoted to propel the identification and development of Ang2 inhibitors and Tie2 agonists [55, 56].

## 5. Conclusion

**Figure 7:**
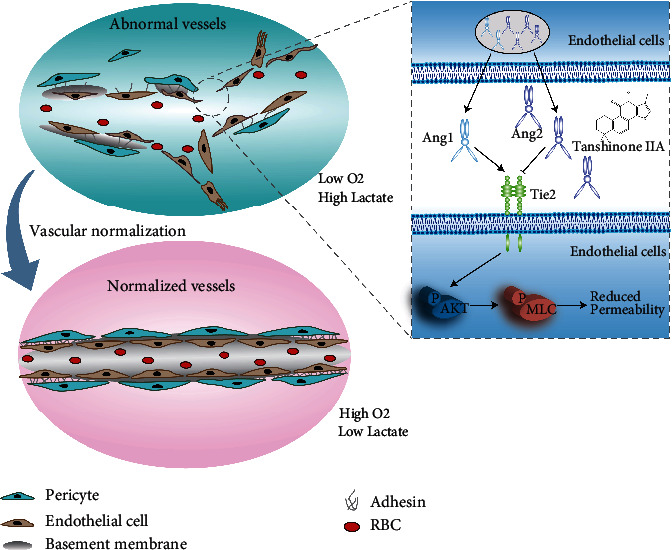
Tan IIA normalizes vessels in tumors and ischemic injury via regulating the Ang2/Tie2 signaling pathway. It revealed that Tan IIA maintained vascular stability by targeting the Ang2-Tie2-AKT-MLCK cascade.

The data presented in this study suggest that Tan IIA plays significant roles in achieving vascular normalization and has a considerable potential for the treatment of ischemic injury and colon cancer. It was further concluded that Tan IIA could suppress the secretion of Ang2 and potentiate the activation of Tie2 signaling in ECs, resulting in decreased vascular permeability and elevated vascular integrity. Mechanistically, we uncovered that Tan IIA maintained vascular stability by targeting the Ang2-Tie2-AKT-MLCK cascade (Figure 7).

## Data Availability

The datasets generated and/or analyzed during the current study are available from the corresponding authors on reasonable request.

## Supplementary Materials

Supplementary Materials Figure S1: Tan IIA improved blood perfusion in the ischemic hind limbs model. Figure S2: Tan IIA caused no notable side effects indicated by body weight and visceral index. Figure S3: Tan IIA promoted vessel normalization in the HT-29 tumors. Table S1: the sequence of primers.
