# Supplementary material for: Targeting the Ang2/Tie2 Axis with Tanshinone IIA Elicits Vascular Normalization in Ischemic Injury and Colon Cancer
Source: Oxid Med Cell Longev. 2021 Nov 10;2021:7037786. doi: 10.1155/2021/7037786 (PMC8598375; doi:10.1155/2021/7037786)
Supplement: Supplementary Materials — Figure S1: Tan IIA improved blood perfusion in the ischemic hind limbs model. Figure S2: Tan IIA caused no notable side effects indicated by body weight and visceral index. Figure S3: Tan IIA promoted vessel normalization in the HT-29 tumors. Table S1: the sequence of primers. [file 7037786.f1.docx]

# Supplementary Materials


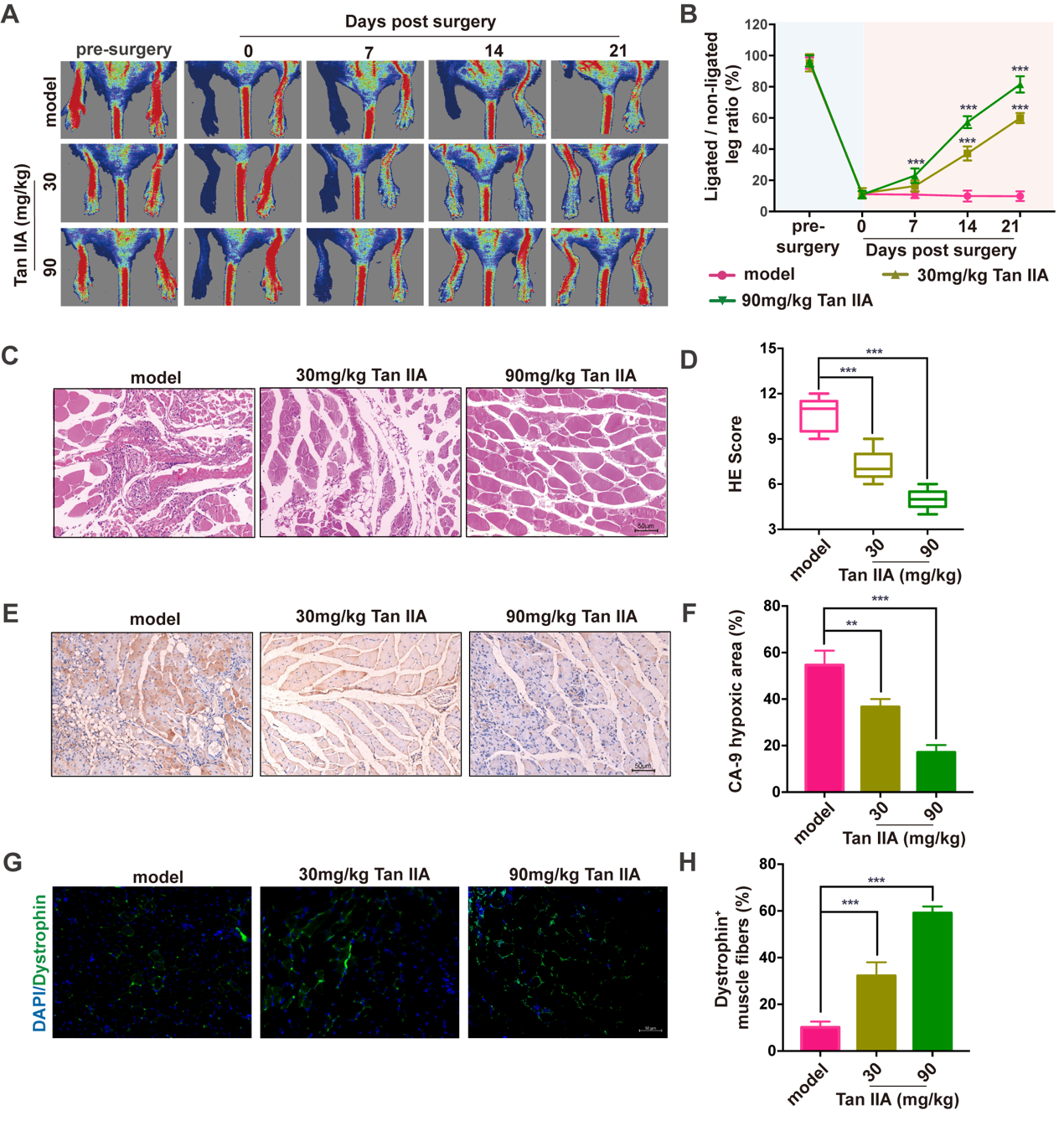


**Figure S1** Tan IIA improved blood perfusion in the ischemic hind limbs model. (A) Representative images of LDPI of the ischemic hind limbs in mice treated with 0.3% CMC-Na or Tan IIA at the indicated time points. (B) Hindlimb blood flow expressed as a percentage of ischemic limb blood flow over nonischemic hindlimb blood flow measured at the indicated time points (n=5). (C) Representative images of H&E staining for the gastrocnemius muscle at 21 days post-surgery. Scale bar, 50 μm. (D) Histological scoring of H&E staining for the mice treated with 0.3% CMC-Na or Tan IIA (n=3). (E) Hypoxia in the gastrocnemius muscle tissues at day 21 post-surgery was measured by CA-9 staining (brown). Representative images are shown. Scale bar, 50 μm. (F) Statistical analysis of CA-9 expression in the gastrocnemius muscle tissues (n=3). (G) Representative immunofluorescence images of Dystrophin (green) to reflect functional muscle fibers in the gastrocnemius muscle tissues are shown. Scale bar, 25 μm. (H). Quantification of Dystrophin expression in the gastrocnemius muscle tissues. The data were presented as mean ± SD. **p<0.05, ***p<0.001 (versus model group).

**Figure S2** Tan IIA caused no notable side effects indicated by body weight and visceral index. (A) Body weight changes of mice in different groups at 1, 5, 9, 13, 17, 21 days post-surgery. (B) The visceral index of mice from different groups at day 21 post-surgery.


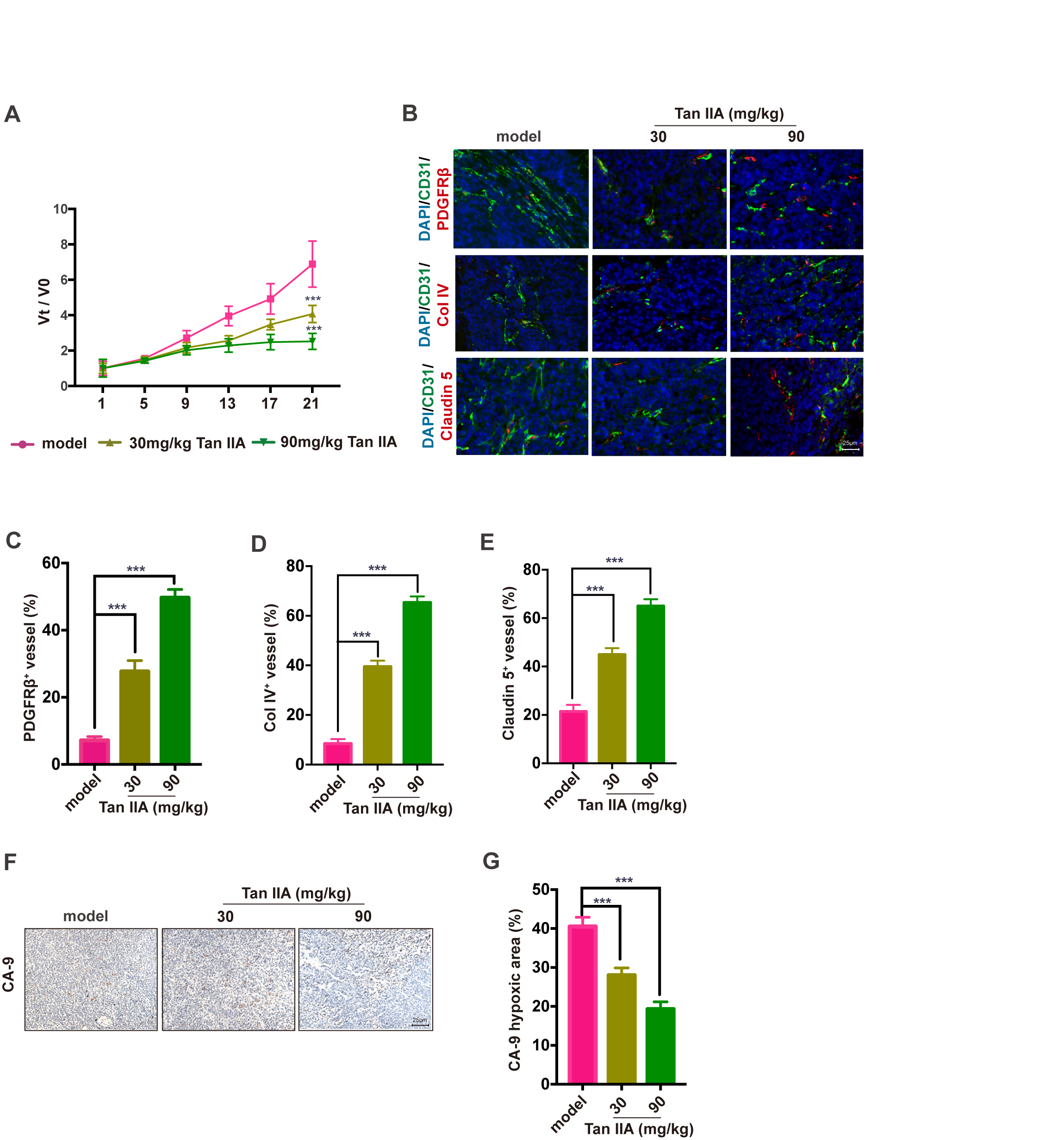


**Figure S3** Tan IIA promoted vessel normalization in the HT-29 tumors. (A) Growth curve of HT-29 tumors in the mice treated with 0.3% CMC-Na and Tan IIA (30 mg/kg and 90 mg/kg) (n=5). (B) Representative immunofluorescence images of PDGFRβ, Collagen IV and Claudin 5 in the tumor blood vessels are shown. Scale bars, 25 μm. (C) Quantification of PDGFRβ expression in the tumor blood vessels (n=3). (D) Quantification of Collagen IV expression in the tumor blood vessels (n=3). (E) Quantification of Claudin 5 expression in the tumor blood vessels (n=3). (F) Hypoxia in the tumor parenchyma was determined by CA-9 staining (brown). Representative immunohistochemical staining images are shown. Scale bar, 25 μm. (G) Quantification of CA-9 expression in the HT-29 tumors (n=3). The data were presented as mean ± SD. *p < 0.05, **p < 0.01, ***p < 0.001 (versus model group).

**Table S1** The sequence of primers

| Gene name | Forward | Reverse |
| --- | --- | --- |
| ANGPT2 | 5’-AACTCCTCTGAAGGCGAAGC-3’ | 5’-AGACCTTGTGCTGGGTGATG-3’ |
| VEGFA | 5’-CAATCGAGACCCTGGTGGAC-3’ | 5’-TCGTTTTTGCCCCTTTCCCT-3’ |
| MMP9 | 5’-GGTGATTGACGACGCCTTTG-3’ | 5’-GGACCACAACTCGTCATCGT-3’ |
| TEK | 5’-AGCCTTCCAAAACGTGAGGG-3’ | 5’-GTTCAGGGGCTTCTCCAGTC-3’ |
| PDGFB | 5’-GCTCTTCCTGTCTCTCTGCTG -3’ | 5’-AGATTGGCTTCTTCCGCACA -3’ |
| Angpt2 | 5’-CGCTGGTGAAGAGTCCAACT -3’ | 5’-CACATGCGTCAAACCACCAG -3’ |
| Vegfa | 5’-CGTCCAACTTCTGGGCTCTT -3’ | 5’-CGCCTTGGCTTGTCACATTTT -3’ |
| Mmp9 | 5’-CAGACGTGGGTCGATTCCAA -3’ | 5’-AACGGGAACACACAGGGTTT -3’ |
| Tek | 5’-AGGCATTCCAGAACGTGAGA -3’ | 5’-CAGTTCAGGGGCTTCTCCAG -3’ |
| Pdgfb | 5’-CACTCCATCCGCTCCTTTGA -3’ | 5’-AGCTTTCCAACTCGACTCCG -3’ |
